# A study on the preparation conditions of lidocaine microemulsion based on multi-objective genetic algorithm

**DOI:** 10.3389/fphar.2023.1272454

**Published:** 2023-09-29

**Authors:** Yuchao Qiao, Xuchun Wang, Hao Ren, Yu Cui, Jiahui Ren, Chongqi Hao, Zhiyang Zhao, Jing Liu, Ruiqing Zhao, Yiting Li, Qingping Tian, Lixia Qiu

**Affiliations:** ^1^ Department of Health Statistics, School of Public Health, Shanxi Medical University, Taiyuan, Shanxi, China; ^2^ School of Pharmacy, Shanxi Medical University, Taiyuan, China

**Keywords:** lidocaine microemulsion, multi-objective optimization, genetic algorithm, Pareto non-inferior solution, non-dominated sorting genetic algorithm-II

## Abstract

**Background:** Topical lidocaine microemulsion preparations with low toxicity, low irritation, strong transdermal capability and convenient administration are urgently needed.

**Methods:** Box-Behnken design was performed for three preparation conditions of 5% lidocaine microemulsions: mass ratio of the mass ratio of surfactant/(oil phase + surfactant) (X_1_), the mass ratio of olive oil/(α-linolenic acid + linoleic acid) (X_2_) and the water content W% (X_3_). Then, five multi-objective genetic algorithms were used to optimize the three evaluation indices to optimize the effects of lidocaine microemulsion preparations. Finally, the ideal optimization scheme was experimentally verified.

**Results:** Non-dominated Sorting Genetic Algorithm-II was used for 30 random searches. Among these, Scheme 2: X_1_ = 0.75, X_2_ = 0.35, X_3_ = 75%, which resulted in Y_1_ = 0.17 μg/(cm^2^·s) and Y_2_ = 0.74 mg/cm^2^; and the Scheme 19: X_1_ = 0.68, X_2_ = 1.42, X_3_ = 75% which resulted in Y_1_ = 0.14 μg/(cm^2^·s) and Y_2_ = 0.80 mg/cm^2^, provided the best matches for the objective function requirements. The maximum and average fitness of the method have reached stability after 3 generations of evolution. Experimental verification of the above two schemes showed that there were no statistically significant differences between the measured values of Y_1_ and Y_2_ and the predicted values obtained by optimization (*p* > 0.05) and are close to the target value.

**Conclusion:** Two lidocaine microemulsion preparation protocols were proposed in this study. These preparations resulted in good transdermal performance or long anesthesia duration, respectively.

## 1 Introduction

Lidocaine is widely used in clinical local anesthetic drugs (Jiamin et al., 2013). Preparation as a surface anesthetic with strong penetrating ability can promote rapid pain relief, overcome the first-pass effect of the liver, and reduce pain following laser cosmetic surgery, dermatological surgery, and puncture examination, resulting in better outcomes. Microemulsion is a novel transdermal drug delivery preparation. Microemulsions are simple to prepare and promote transdermal drug absorption, improve drug solubility and stability, and reduce skin irritation (Tian et al., 2012). Microemulsions are the preferred type of preparation for local anesthetic drugs. Microemulsions are transparent or translucent, thermodynamically stable colloidal systems formulated with an oil phase, water, surfactant, and cosurfactant (Callender et al., 2017). Preparation of microemulsions generally requires a chemically stable oil phase, in which the drug is soluble. In addition, microemulsions must support penetration and association of the drug with the surfactant molecules (Heuschkel et al., 2008). Long chain triglycerides of vegetable origin such as hemp oil, cottonseed oil, and soybean oil are commonly used oil phases. Surfactants, also known as emulsifiers or amphoteric compounds, play an important role in formation of microemulsions through their ability to solubilize and reduce interfacial tension between the oil and water phases (Brosig et al., 2023). Surfactants can be classified as nonionic and ionic. Commonly used surfactants are lecithin (phosphatidylcholine), sodium deoxycholate (bile salt), polyoxyethlene sorbitan monolaurate (Tween 20, 40, 60, 80) and sorbitan monolaurate (Span 20, 40, 60, 80) (Bonferoni et al., 2019). Cosurfactant further reduce interfacial tension, and increase the fluidity of the interface, thereby increasing the entropy of the system. Cosurfactants may also adjust the curvature of the interfacial film by partitioning between the tails of the surfactant chains, allowing greater penetration of the oil between the surfactant tails (Flanagan and Singh, 2006; Golwala et al., 2020). Medium- or short-chain alcohols such as ethanol, ethylene glycol, propylene glycol and polyethylene glycol are commonly used as cosurfactants (Zhang et al., 2014).

Selecting the right surfactant and cosurfactant is a major step in designing a microemulsion system. However, surfactants and cosurfactant have potential to irritate or corrode tissues (Lawrence and Rees, 2000). Many surfactants irritate the skin through their relative ability to dissolve lipid membranes (Effendy and Maibach, 1995). A study by Fitsum F. Sahle showed that increased surfactant content resulted in increased epidermal irritation (Sahle et al., 2014). Therefore, reduction of surfactant content is important in topical lidocaine microemulsions to promote low toxicity, low irritation, strong transdermal properties, and convenient administration. The following key findings have driven microemulsion development: (1) Use of mixed oil as the oil phase can significantly increase the microemulsion area, thereby reducing the amount of surfactant used in the preparation process (You et al., 2014); (2) The method of mixing surfactant can effectively improve the solubility of surfactant, thus increasing the area of microemulsion, allowing for use of less surfactant (Sun et al., 2014); (3) The microemulsion area of olive oil (OL) is larger in the absence of cosurfactant than in the presence of cosurfactant under the action of mixed surfactant, and vitamin E succinate (VES) can further increase the area of microemulsion of OL (Jing et al., 2019). Based on the above research basis, the group, Prof. Tian, conducted a research on the preparation process of low surfactant, co-surfactant free lidocaine microemulsion with vitamin E succinate assisted mixed oil phase.

During the development process, we evaluated the effectiveness of lidocaine microemulsion preparation using the following three indicators: steady-state penetration rate, skin retention and microemulsion particle size. Steady-state permeation rate and skin retention are two important parameters related to transdermal drug delivery, which are commonly used to study and evaluate drug penetration and absorption in the skin. Among them, steady state permeation rate is the rate at which a drug passes through the membrane or interface of an organism or drug delivery system to reach a steady state. This parameter is important for determining efficiency and control of drug transdermal delivery because it can be used to estimate the rate of drug absorption in the skin. Skin retention refers to the amount of drug that remains in skin tissue after a certain period of time. This parameter is used to evaluate the residence time of drugs on the skin. The amount of skin retention is crucial for evaluation of local treatment and skin irritation. Therefore, this study required multi-objective optimization (Bansal et al., 2021) to optimize microemulsion preparation conditions for the three objectives simultaneously. However, traditional multi-objective optimization methods such as contour plots (Li et al., 2011; Patel et al., 2014), multi-objective weighting (Horn et al., 1994; Augusto et al., 2006), goal programming (Horn et al., 1994; Tamiz et al., 1998; Corne et al., 2000), the constraint method (Haimes et al., 1971) and the minimax method (Li and Li, 1996) can only provide an unique optimal solution. However, these approaches violate the principle of multi-objective optimization and is highly subjective in assigning target weights. Therefore, we need to coordinate and compromise among the sub-objectives to optimize each sub-objective (Xunxue, 2006).The genetic algorithm approach (Holland, 1975; Farhang-Mehr et al., 2002) is suitable for optimizing complex multi-objective, nonlinear systems with good global search performance. Genetic algorithms can obtain a set of solutions in each run such that each target is optimal, and no other solution in the search space is superior. This result is called the Pareto non-inferior solution set (De Jong, 1975; Konak et al., 2006; Xunxue, 2006; Bansal et al., 2021). As a result, researchers can be provided with a variety of alternative, uncontrolled optimal solutions.

In this study, five multi-objective genetic algorithms were used to optimize the preparation conditions for a lidocaine microemulsion. The optimization effects of the five genetic algorithms were compared to select the relatively optimal preparation scheme, and the most ideal scheme was experimentally verified to determine the optimal preparation conditions of a lidocaine microemulsion formulation.

## 2 Materials and methods

### 2.1 Materials

The test materials and chemical sources are detailed in the paper “Design, optimization and evaluation of cosurfactant free microemulsion-based hydrogel with low surfactant for enhanced transdermal delivery of lidocaine” published in International Journal of Pharmaceutics by our group in August 2020 (Zhang et al., 2020).

### 2.2 Preparation of lidocaine microemulsions

The lidocaine microemulsion is comprised of lidocaine 5% (w/w), Alpha-linolenic acid (ALA), linoleic acid (LA), OL, VES, Cremophor RH40, sorbitan monooleate 80 (Span 80), and water.

Preparation of mixed oil phase (O) was performed as follows: ALA and LA were mixed at a 1:4 ratio. This mixture was then mixed with OL at 1:4, 1:1, and 4:1 to obtain mixed oils O_1_, O_2_ and O_3_, respectively. Vitamin E succinate was used as the auxiliary oil, and complexes of O_1_-VES, O_2_-VES and O_3_-VES (6:1 w/w) were used as the oil phase, which significantly increased the microemulsion area, allowing for reduced amount of surfactant.

Preparation of mixed surfactant (S) was performed as follows: RH40 and Span 80 were mixed at a 5:1 (w/w) ratio. This allowed for improved the solubility of the surfactant, increased microemulsion area, and reduced the amount of surfactant.

Microemulsions containing lidocaine were prepared by dissolving lidocaine 5% (w/w) in a mixture of different proportions of O and S at room temperature, then slowly adding the appropriate amount of water with magnetic stirring.The mass ratio of mixed oil phase to mixed surfactant, the weight ratio of oil phase mixing, and the water content as lidocaine microemulsion drug delivery system were determined based on our previous study. These factors were expressed as the mass ratio of S/(O + S) (X_1_), the weight ratio of OL/(ALA + LA) (X_2_), and the water content W% (X_3_), respectively.

A 3-factor, 3-level Box-Behnken design was used for the above factors, with a total of 15 test protocols, each replicated six times. The levels of X_1_, X_2_, and X_3_ were 0.6–0.8, 0.25–4, and 65%–75%, respectively. Steady-state permeation rate (Y_1_), skin retention (Y_2_), and microemulsion particle size (Y_3_) were used as evaluation indices to evaluate the effects of the preparation process. A larger Y_1_ value results in better microemulsion transdermal properties, and a larger Y_2_ value results in longer duration of anesthetic effects. The Y_3_ must be less than 100 nm to guarantee the transdermal properties of the microemulsion. The steady-state permeability, skin retention, and particle size of the lidocaine microemulsions are shown in Table 1. Although the microemulsion particle size (Y3) met the requirement of less than 100 nm in all 15 protocols, the steady-state permeation rate (Y_1_) and skin retention (Y_2_) were not simultaneously optimal in the test results. Therefore, the preparation process conditions must be optimized using mathematical modeling combined with a multi-objective optimization approach to optimize Y_1_ and Y_2_ simultaneously.

**TABLE 1:** Box-Behnken design scheme and results for lidocaine microemulsion formulation.

Solutions	X_1_	X_2_	X_3_ (%)	Y_1_ (mg/(cm^2^·s))	Y_2_ (mg/cm^2^)	Y_3_ (nm)
1	0.8	0.25	70	0.1568 ± 0.003	0.2629 ± 0.026	17.83 ± 3.668
2	0.8	1	65	**0.1878 ± 0.035**	0.4089 ± 0.015	13.64 ± 0.340
3	0.8	1	75	0.1498 ± 0.019	0.6672 ± 0.340	16.31 ± 1.849
4	0.8	4	70	0.0907 ± 0.012	0.2670 ± 0.035	23.21 ± 0.594
5	0.7	0.25	65	0.1098 ± 0.038	0.3216 ± 0.045	20.13 ± 0.109
6	0.7	0.25	75	0.1034 ± 0.039	0.8754 ± 0.072	22.98 ± 0.390
7	0.7	4	65	0.0986 ± 0.027	0.2787 ± 0.028	19.86 ± 0.247
8	0.7	4	75	0.1226 ± 0.021	**1.0292 ± 0.376**	22.70 ± 0.376
9	0.6	0.25	70	0.1395 ± 0.035	0.3090 ± 0.182	42.38 ± 0.182
10	0.6	1	75	0.1279 ± 0.011	0.6596 ± 0.025	41.36 ± 0.157
11	0.6	1	65	0.0893 ± 0.009	0.5231 ± 0.071	36.35 ± 0.183
12	0.6	4	70	0.0902 ± 0.024	0.4388 ± 0.139	41.39 ± 0.149
13	0.7	1	70	0.1527 ± 0.029	0.3586 ± 0.094	20.78 ± 0.183
14	0.7	1	70	0.1001 ± 0.003	0.3167 ± 0.032	20.81 ± 0.132
15	0.7	1	70	0.1062 ± 0.009	0.2928 ± 0.026	21.31 ± 0.128

Note: Bold indicates the most ideal result. The values were expressed as Mean ± SD (*n* = 6).

### 2.3 Methods of model building

Since the microemulsion particle sizes were all less than 100 nm, Y_3_ was no longer modeled. Steady-state permeation rate (Y_1_) and skin retention (Y_2_) were used as dependent variables, and the mass ratio of S/(O + S) (X_1_), weight ratio of OL/(ALA + LA) (X_2_), and W% (X_3_) were used as independent variables. Quadratic polynomial models were developed for Y_1_ and Y_2_ as the objective functions for the multi-objective optimization of process conditions.

The fit of the model is determined by the coefficient of determination (*R* ^2^). *R* ^2^ is an indicator for evaluating the effectiveness of model fitting, which estimates how well the fitted model fits the observed values. An *R* ^2^ of 1 indicates that the predicted values of the model exactly match the actual values, and values closer to 1 indicate high accuracy.

The model expressions are as follows:

$$\hat{y}={\hat{\beta }}_{0}+\sum_{i=1}^{m}{\hat{\beta }}_{i}x_{i}+\sum_{i=1}^{m}{\hat{\beta }}_{i}{x_{i}}^{2}+\sum_{i=1}^{m}\sum_{j=1}^{m}{\hat{\beta }}_{ij}x_{i}x_{j}$$

(i < j, m is the number of factors)

### 2.4 Multi-objective genetic algorithms

A quadratic polynomial full model established by Y_1_ and Y_2_ are set as the objective functions of multi-objective optimization, and the two objective functions are set to be maximized. The ranges of the three factors were set as X_1_: 0.6–0.8, X_2_: 0.25–4.0 and X_3_: 65%–75%. Five multi-objective genetic algorithms, detailed in Sections 2.4.1–2.4.5, were used to optimize the drug delivery system.

#### 2.4.1 Vector evaluated genetic algorithm

Vector Evaluated Genetic Algorithm (VEGA) (Schaffer, 1985; Xunxue and Chuang, 2005; Dias and De Vasconcelos, 2002) is a population-based non-Pareto method (Dan and Rui, 2004). This method uses a proportional selection mechanism, which is superior to a single-objective algorithm. The principle of VEGA is to generate a corresponding subpopulation for each sub-objective function. If the number of sub-objectives of a multi-objective problem is k, the population needs to be randomly and equally divided into k subpopulations of equal size, where the size of each subpopulation is N/k (N is the size of entire population). Each sub-objective function completes selection, evaluation, and operation in its corresponding subpopulation independently, then forms a new group for crossover and variation operation. Therefore, the process of “splitting, juxtaposition, evaluation, selection, and merging”is executed in a cycle, resulting in a non-inferior solution to the problem.

#### 2.4.2 Multiple objective genetic algorithm

Multiple Objective Genetic Algorithm (MOGA) (Fonseca and Fleming, 1993) ranks each individual in the population using the concept of “Pareto Optimal Individuals”, so that the best individuals values in the population have a greater chances to be inherited by the next-generation population. Following a specified number of generations of cycles, the optimal solution of the multi-objective optimization problem can finally be searched. The algorithm flow of MOGA is as follows: an initial population *p* with sample number N is randomly generated. After the non-dominated sorting, the first generation population Q is generated by cross-variance and other operations. Then, the parent population *p* is merged with the child population Q to generate the second generation population, and the non-dominated set is constructed by non-dominated sorting and calculating the distance between individuals. A new population is generated by crossover and mutation, and the cycle is repeated until the termination condition is satisfied. Finally, the distance between individuals is calculated, and the optimal alternative is obtained by reordering the fitness according to the base.

#### 2.4.3 Niched pareto genetic algorithm

Niched Pareto Genetic Algorithm (NPGA) (Fonseca and Fleming, 1993; Horn et al., 1994) uses a tournament selection mechanism to select the best individuals for subsequent evolutionary reproduction, while incorporating niche technique to maintain the diversity and homogeneity of the distribution of individuals in the candidate solution set. The selection mechanism of NPGA is a combination of tournament selection and external auxiliary selection. After obtaining the initial population, 10 individuals are randomly selected to form the external comparison set CS. In addition, two individuals, p_i_ and p_j_, are randomly selected to make a two-by-two comparison with the above 10 individuals. If one of these is superior to the comparison set and the other is inferior to the comparison set, the former is selected and copied. If both randomly selected individuals are superior or inferior to the external comparison set, the better of the two is selected using the sharing mechanism. That is, according to the number of niches, focus on selecting individuals with the smallest number of niche for replication to obtain the optimal solution with uniform distribution on the front end.

#### 2.4.4 Non-dominated sorting genetic algorithm

Non-dominated Sorting Genetic Algorithm (NSGA) (Srinivas and Deb, 1994) uses non-dominated sorting as the solution sorting criterion, and the fitness is reasonably assigned to a diverse search space (Heris et al., 2011). In this approach, all individuals are graded at different levels, and before performing the selection operator, the population is graded and sorted according to dominance and non-dominance relationships. To maintain population diversity, all individuals in the population are assigned a virtual fitness value (generally proportional to the population size), and individuals at the same level have the same virtual fitness value, thus ensuring that individuals at the same level have the same probability of replication. This group of graded individuals is then ignored, and the other individuals in the population are graded again according to the dominance non-dominance relationship and given new virtual fitness values that are less than the values in the previous level. This operation is repeated for the remaining individuals until all individuals in the population are graded.

#### 2.4.5 Non-dominated sorting genetic algorithm-II

Non-dominated Sorting Genetic Algorithm-II (NSGA-II) (Deb et al., 2000) greatly reduces computational complexity by introducing a fast non-dominated sorting algorithm, using an elite strategy design to increase the sample space, and using individual crowding and crowding comparison operators as grading criteria. This can provide a multi-objective optimized Pareto non-inferiority solution set and ensure the diversity of the population. The algorithm process of NSGA-II is as follows. First, the parent population P_n_ is used to generate the child population Q_n_, and the two populations are combined to form a population R_n_ of size 2n. A non-inferiority classification operation is performed on this population. Then, the crowding degree of all individuals in each non-inferior class is calculated. The next-generation population P_n+1_ is generated according to the principle of crowding selection operator, and the number of evolutionary generations is n+1. Then, whether the number of evolutionary generations of this population is greater than the maximum number of evolutionary generations is determined. If yes, then the algorithm ends, otherwise it continues to evolve. The algorithm continues to evolve until it reaches the maximum number of evolutionary generations specified.

### 2.5 Optimization parameters

The parameters of the five multi-objective genetic algorithms were set as follows: the initial population was 30, the probability crossover was 0.8, the probability of mutation was 0.05, the maximum evolutionary generation was 100, and 30 random searches were performed to give the Pareto non-inferiority solution set.

### 2.6 Measurement of steady-state permeation rate, skin retention, and particle size

The abdominal skin (free of subcutaneous fat and adherent tissue) of guinea pigs was rinsed with saline and fixed with the horned layer facing upward between the supply and receiving cell. We added 1.0 g of each formulation was placed on the skin surface of the supply cells, and 15 mL of the receiving solution consisting of phosphate buffered saline and ethanol was placed under a magnetic rotor (set to 350 rpm) in the receiving cells. The temperature was maintained at 37°C ± 0.1°C. Samples (2.0 mL) were taken at predetermined time points (0.5, 1.0, 2.0, 4.0, 6.0, 8.0, 10.0, and 12.0 h), and filtered through 0.45-µm microporous filtration membrane. The cells were then replenished with an equal amount of fresh receiving solution. The filtrates were analyzed using high performance liquid chromatography (HPLC), the peak areas were determined, and the corresponding drug concentrations were calculated. The cumulative permeation of the drug was then calculated based on the transdermal diffusion area, the drug concentration at different time points, and the volumes of the receiving pool and the sampling volume. The slope of the linear regression curve of the cumulative permeation of the drug against t (h) represents the steady-state permeation rate.

After the skin permeability measurement was completed, the skin was rinsed with fresh receiving solution. The skin was dried on filter paper, then cut and soaked in methanol (4 mL) for 24 h to fully extract the drug remaining in the skin. The concentration of lidocaine in the supernatant was then analyzed using HPLC to determine skin retention.

The microemulsion was diluted 50-fold with distilled water and particle size was determined using a Nano Zetasizer (ZS90, Malvern Instruments, Worcestershire, UK).

### 2.7 Statistical software

Model establishment and statistical analysis of optimization results were performed using SPSS 22.0. Multi-objective genetic algorithm optimization was performed using a Matlab 2009a plug-in SGALAB toolbox bete5008 written by a member of the group, Chen, a software engineer at the University of Glasgow, United Kingdom. The average level of Pareto non-inferiority solutions and objective function values were expressed as the median and interquartile range.

## 3 Results

### 3.1 Quadratic polynomial model and model fitting results

The quadratic polynomial full model and model fit results for steady-state infiltration rate (Y_1_) and skin retention (Y_2_) are shown below. The *R* ^2^ value of the two models were 0.6537 and 0.7264, respectively, which indicated that the respective variables could explain 65.4% and 72.6% of the variation in Y_1_ and Y_2_, respectively. The explanations of the respective variables of the models are high, and the models are well fitted.

$$\hat{Y_{1}}=0.921+2.312X_{1}+0.041X_{2}-5.138X_{3}+0.442{X_{1}}^{2}+0.005{X_{2}}^{2}+5.847{X_{3}}^{2}-0.044X_{1}X_{2}-3.829X_{1}X_{3}-0.062X_{2}X_{3}\;R^{2}=0.6537$$

$$\hat{Y_{2}}=54.716-0.722X_{1}-0.629X_{2}-057.078X_{3}-3.039{X_{1}}^{2}+0.004{X_{2}}^{2}+110.563{X_{3}}^{2}-0.174X_{1}X_{2}+6.889X_{1}X_{3}+1.072X_{2}X_{3}\;R^{2}=0.7264$$

### 3.2 Multi-objective genetic algorithm optimization results

The random search results, fitness evolution algebra, and optimal non-inferiority solution schemes of VEGA, MOGA, and NSGA were inferior to those obtained using NPGA and NSGA-Ⅱ. To remain concise, only the random search results and maximum fitness and average fitness evolution curves from NPGA and NSGA-Ⅱ, which have relatively better optimization effects, are listed in this manuscript. The optimization results of the remaining three methods are summarized in the appendix.

Our primary criterion for determining the most desirable regimen is that the regimen results in a relatively maximum steady-state penetration rate, and skin retention. On the basis of the above we would like to use less surfactant. Additionally, based on these two criteria, the amount of material in the formulation would be relatively small.

#### 3.2.1 NPGA optimization results

The non-inferior solution schemes obtained from the NPGA random search (30 iterations) are shown in Table 2. According to the requirements for the objective function, the optimal process conditions were selected in the Pareto solution set with X_1_ = 0.68, X_2_ = 0.89, and X_3_ = 75% for Scheme 20. The steady-state permeation rate and skin retention of lidocaine microemulsion were Y_1_ = 0.15 mg/(cm^2^·s) and Y_2_ = 0.77 mg/cm^2^.

**TABLE 2:** NPGA random search results.

Solutions	Pareto optimal solution set			Response		Surfactant (%)
	X_1_	X_2_	X_3_ (%)	Y_1_ (μg/(cm^2^·s))	Y_2_ (mg/cm^2^)	
1	0.79	2.32	75	0.12	0.82	15.8
2	0.67	1.07	75	0.15	0.75	13.4
3	0.75	0.28	74	0.17	0.64	15.8
4	0.63	1.85	75	0.13	0.81	12.6
5	0.77	1.53	74	0.14	0.70	16.2
6	0.67	2.08	74	0.12	0.71	14.1
7	0.64	1.05	74	0.14	0.61	13.4
8	0.78	0.65	75	0.16	0.69	15.6
9	0.63	1.06	74	0.14	0.62	13.2
10	0.60	0.62	75	0.16	0.69	12.0
11	0.62	2.06	75	0.13	0.82	12.4
12	0.68	3.62	74	0.11	0.81	14.3
13	0.77	2.88	75	0.12	0.85	15.4
14	0.74	2.65	73	0.11	0.63	16.3
15	0.68	1.21	75	0.14	0.72	13.6
16	0.60	0.44	75	0.17	0.70	12.0
17	0.68	1.49	75	0.13	0.75	13.6
18	0.70	1.06	74	0.14	0.69	14.7
19	0.72	0.65	74	0.15	0.62	15.1
**20**	**0.68**	**0.89**	**75**	**0.15**	**0.77**	**13.6**
21	0.68	1.93	73	0.12	0.63	15.0
22	0.79	4.00	74	0.11	0.80	16.6
23	0.61	0.72	75	0.15	0.68	12.2
24	0.74	0.53	75	0.16	0.73	14.8
25	0.66	1.23	75	0.14	0.74	13.2
26	0.79	1.58	74	0.13	0.60	16.6
27	0.60	1.75	75	0.13	0.74	12.0
28	0.77	3.26	74	0.11	0.76	16.2
29	0.62	3.55	74	0.12	0.81	13.0
30	0.77	2.48	75	0.12	0.80	15.4

Note: Bold indicates the most ideal solution for this method.

**FIGURE 1:**
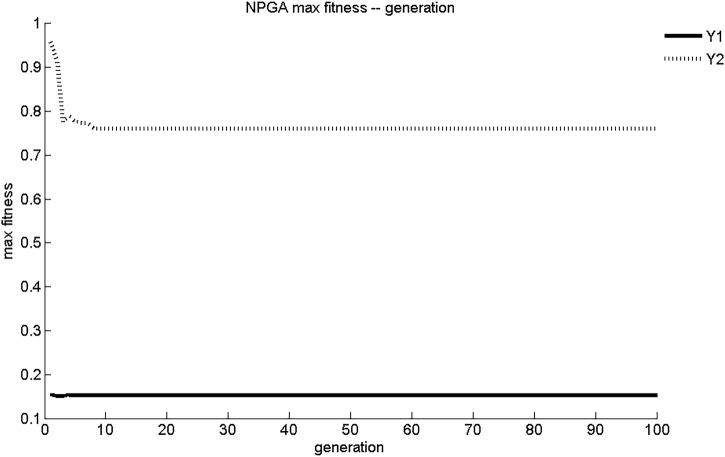
NPGA maximum adaptation evolutionary curve.

**FIGURE 2:**
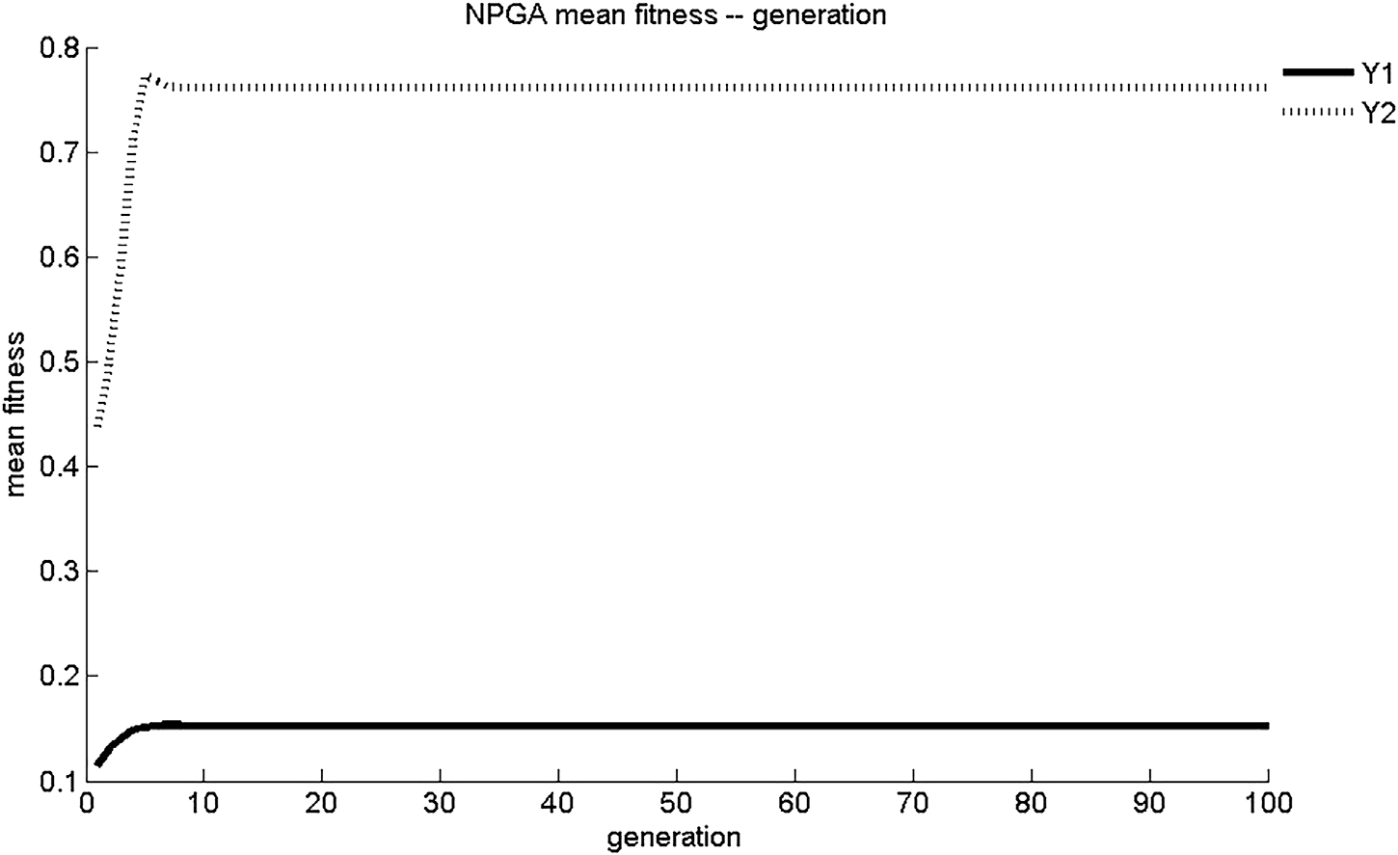
NPGA average adaptation evolutionary curve.

The maximum fitness and average fitness evolution curves of the two sub-objective functions obtained using NPGA optimization are shown in Figures 1, 2. The maximum fitness evolution curve is used to evaluate the convergence performance of the algorithm, reflecting the change in the solution. This is a measure of whether the algorithm can find the global optimal solution to the problem with infinite iterations. As shown in Figure 1, NPGA found the maximum fitness for Y_1_ and Y_2_ only after 7 generations of evolution. These values were 0.153 mg/(cm^2^·s) and 0.761 mg/cm^2^, respectively. The convergence of NPGA was general. The average fitness evolution curve was used to evaluate the dynamic performance of the algorithm, reflecting the change of the objective function with the best fitness and the largest value in each generation with the number of evolutionary generations. As shown in Figure 2, the objective function in the initial generations had lower adaptation and smaller objective function values. As the number of evolutionary generations increased, the fitness of the objective function increased rapidly and the objective function value increased. There was a slight degradation around five generations of evolution. After about seven generations of evolution, the values of Y_1_ and Y_2_ were stable at 0.15 mg/(cm^2^·s) and 0.76 mg/cm^2^, respectively. The dynamic performance of NPGA was average.

#### 3.2.2 NSGA-Ⅱ optimization results

The non-inferior solution schemes obtained from 30 random searches using NSGA-II are shown in Table 3. According to the requirements for the objective function, the optimal process conditions were Scheme 2 with X_1_ = 0.75, X_2_ = 0.35, and X_3_ = 75% in the Pareto solution set. The corresponding steady-state permeation rate and skin retention of lidocaine microemulsion were Y_1_ = 0.17 mg/(cm^2^·s) and Y_2_ = 0.74 mg/cm^2^, respectively. Scheme 19, with X_1_ = 0.68, X_2_ = 1.42, and X_3_ = 75% also presented optimal process conditions to obtain Y_1_ = 0.14 mg/(cm^2^·s) and Y_2_ = 0.80 mg/cm^2^.

**TABLE 3:** NSGA-II random search results.

Solutions	Pareto optimal solution set			Response		Surfactant (%)
	X_1_	X_2_	X_3_ (%)	Y_1_ (mg/(cm^2^·s))	Y_2_ (mg/cm^2^)	
1	0.78	0.43	75	0.17	0.74	15.6
**2**	**0.75**	**0.35**	**75**	**0.17**	**0.74**	**15.0**
3	0.63	0.57	75	0.16	0.73	12.6
4	0.68	1.84	74	0.12	0.65	14.3
5	0.64	0.98	75	0.15	0.76	12.8
6	0.69	1.37	74	0.14	0.71	14.5
7	0.80	0.96	73	0.15	0.49	17.6
8	0.79	0.39	75	0.17	0.73	15.8
9	0.79	0.29	75	0.18	0.72	15.8
10	0.66	0.38	75	0.17	0.73	13.2
11	0.63	1.81	72	0.11	0.50	14.5
12	0.75	1.13	75	0.15	0.78	15.0
13	0.63	1.04	75	0.15	0.77	12.6
14	0.70	0.75	75	0.16	0.76	14.0
15	0.77	0.30	74	0.17	0.64	16.2
16	0.75	1.41	74	0.14	0.70	15.8
17	0.62	2.21	75	0.13	0.85	12.4
18	0.60	3.94	75	0.13	1.01	12.0
**19**	**0.68**	**1.42**	**75**	**0.14**	**0.80**	**13.6**
20	0.69	0.46	74	0.16	0.66	14.5
21	0.68	1.28	75	0.14	0.80	13.7
22	0.61	0.40	74	0.16	0.63	12.8
23	0.68	0.70	74	0.15	0.67	14.3
24	0.74	1.34	75	0.14	0.79	14.8
25	0.61	0.43	75	0.17	0.71	12.2
26	0.67	3.79	75	0.12	0.99	13.4
27	0.65	0.32	75	0.17	0.72	13.0
28	0.62	2.06	74	0.13	0.74	13.0
29	0.66	0.49	75	0.16	0.74	13.2
30	0.72	0.79	74	0.15	0.67	15.1

Note: Bold indicates the most ideal solution for this method.

**FIGURE 3:**
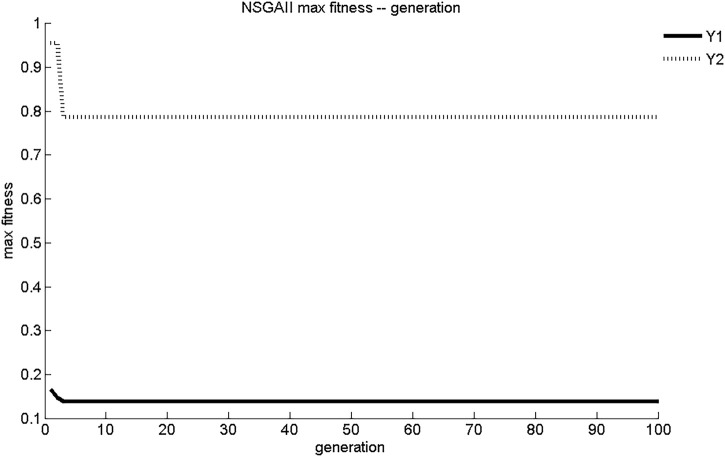
NSGA-II maximum adaptation evolutionary curve.

**FIGURE 4:**
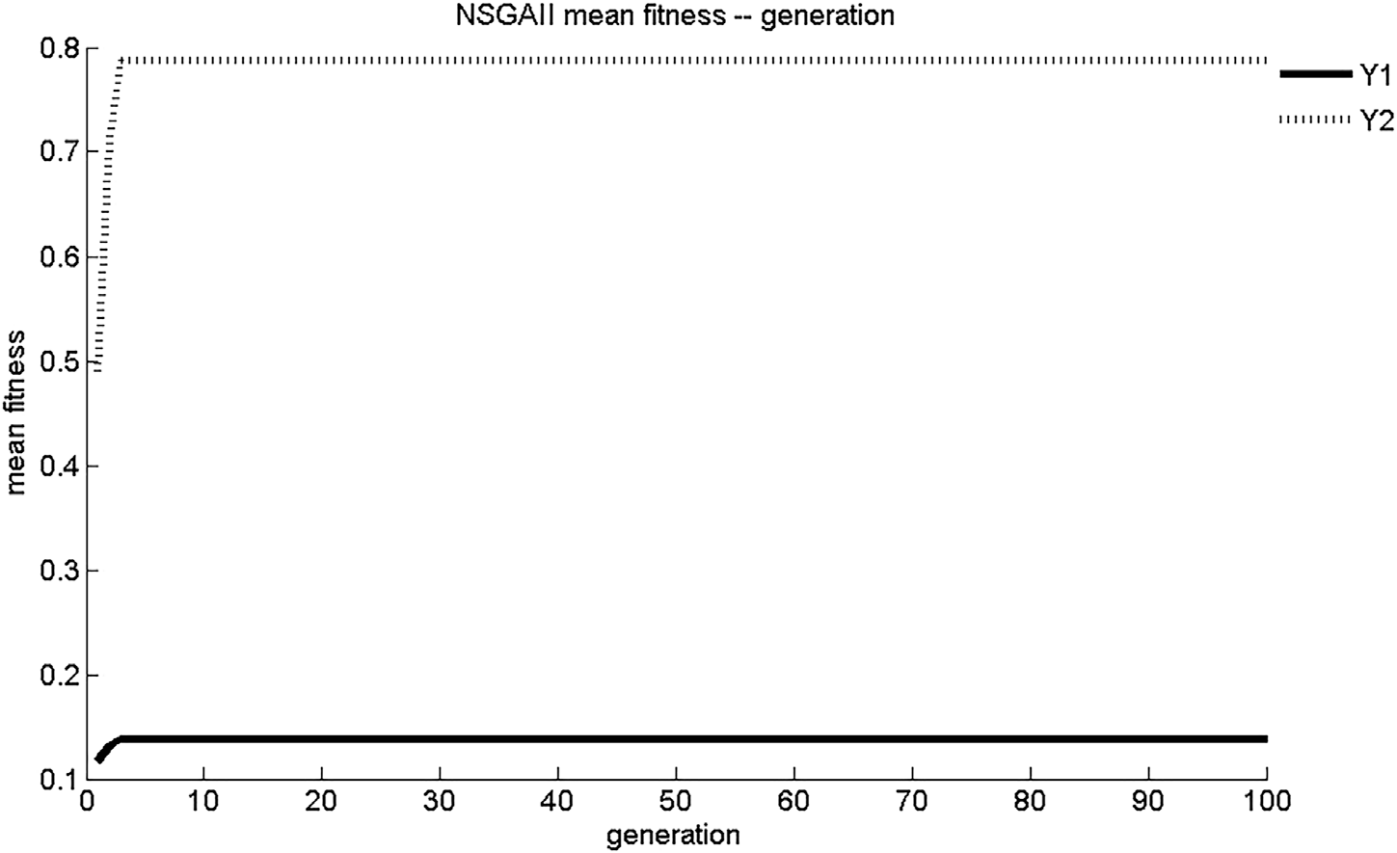
NSGA-II average adaptation evolutionary curve.

The evolution curves of maximum fitness and average fitness of the two sub-objective functions obtained by NSGA-II search are shown in Figures 3, 4. As shown in Figure 3, NSGA-II found the maximum fitness of Y_1_ and Y_2_ at the third generation of evolution, with results of 0.139 mg/(cm^2^·s) and 0.788 mg/cm^2^, respectively. The convergence of NSGA-II was better. As shown in Figure 4, the initial generations of objective function had lower adaptation and smaller objective function values. As the number of generations of evolution increased, the fitness of the objective function increases rapidly and the value of objective function increased. After three generations of evolution, the values of Y_1_ and Y_2_ stabilized at 0.14 mg/(cm^2^·s) and 0.79 mg/cm^2^, respectively. The dynamic performance of NSGA-II was better.

### 3.3 Comparison of five multi-objective genetic algorithms for ideal non-inferior solution schemes

According to the objective requirements of Y_1_ and Y_2_, the objective function values, maximum fitness, and average fitness evolutionary generations corresponding to the most ideal Pareto non-inferiority solution schemes in the set of five multi-objective genetic algorithms Pareto non-inferiority solutions were determined as shown in Table 4. Scheme 2 of NSGA-II: X_1_ = 0.75, X_2_ = 0.35, X_3_ = 75%; Scheme 19 of NSGA-II: X_1_ = 0.68, X_2_ = 1.42, X_3_ = 75%; and NPGA Scheme 20: X_1_ = 0.68, X_2_ = 0.89, X_3_ = 75% were better fits than optimal schemes determined using VEGA, MOGA, and NSGA. However, compared with NPGA, which evolved for seven generations to reach stability in maximum and average fitness, NSGA-II only required three generations, and the convergence and dynamics of the algorithm were better. The ideal Pareto solution schemes of MOGA, VEGA, and NSGA did not match the preparation process requirements and were prone to early convergence, with poor local search capability and dynamics.

**TABLE 4:** Comparison of five multi-objective genetic algorithms for ideal non-inferior solution schemes.

Methods	Solutions	Pareto optimal solution set			Response		Maximum fitness	Average fitness
		X_1_	X_2_	X_3_ (%)	Y_1_ (mg/(cm^2^·s))	Y_2_ (mg/cm^2^)		
VEGA	29	0.65	0.83	75	0.15	0.75	9	9
MOGA	6	0.75	0.99	75	0.15	0.70	11	11
**NPGA**	**20**	**0.68**	**0.89**	**75**	**0.15**	**0.77**	**7**	**7**
NSGA	1	0.71	0.29	65	0.14	0.42	8	8
**NSGA-II**	**2**	**0.75**	**0.35**	**75**	**0.17**	**0.74**	**3**	**3**
	**19**	**0.68**	**1.42**	**75**	**0.14**	**0.80**		

Note: Bold indicates the relatively ideal scheme among the five approaches.

### 3.4 Comparison of the search performance of five multi-objective genetic algorithms

Most of the Pareto non-inferior solutions and objective function values of the five multi-objective genetic algorithms did not have normal distributions. Therefore, the median (M) and interquartile range (IQR) were used to describe the center and variance of the solutions and objective values (Table 5). The median levels of the sub-objective function values obtained by NSGA-II optimization were $M_{Y_{1}}$ = 0.15 and $M_{Y_{2}}$ = 0.73, which were higher than the median levels of the other four methods. The interquartile ranges for NSGA-II optimization were $IQR_{Y_{1}}$ = 0.03 and $IQR_{Y_{2}}$ = 0.10, respectively, and the variability was relatively small, and the search accuracy was better than was observed using the other four algorithms. The medians of the Pareto non-inferior solutions obtained by NSGA-II random search were $M_{X_{1}}$ = 0.68, $M_{X_{2}}$ = 0.88, and $M_{X_{3}}$ = 75.00, respectively, X_1_ and X_2_ were lower than the medians of the other four methods. These results show that NSGA-II could minimize surfactant use in the oil phase and reduced toxicity and irritation of the microemulsions. It also reduced the amount of olive oil used, which represents a cost benefit. Compared with MOGA and NSGA, the median level of X_3_ of Pareto non-inferior solution of NSGA-II was higher, which agreed with the results from VEGA and NPGA. The higher water content has the advantage of improving the transdermal properties of the microemulsion in the oil-water mixed state. The interquartile ranges of the Pareto non-inferiority solutions of NSGA-II were $IQR_{x_{1}}$ = 0.12, $IQR_{x_{2}}$ = 0.99 and $IQR_{x_{3}}$ = 1.00, which were relatively low. The non-inferiority solutions had good stability.

**TABLE 5:** The average level of objective function values and the Pareto non-inferiority solutions of five multi-objective genetic algorithm.

Methods	Variables	Median	P_25_	P_75_	IQR	Test of normality	
						W	*p*
VEGA	X_1_	0.70	0.66	0.74	0.08	0.97	0.58
	X_2_	1.13	0.63	1.63	1.00	0.86	<0.001
	X_3_	75.00	74.00	75.00	1.00	0.67	<0.001
	Y_1_	0.14	0.13	0.16	0.03	0.94	0.08
	Y_2_	0.73	0.67	0.75	0.08	0.83	<0.001
MOGA	X_1_	0.71	0.65	0.75	0.10	0.96	0.30
	X_2_	2.33	0.99	3.11	2.12	0.94	0.09
	X_3_	71.00	68.00	73.00	5.00	0.94	0.08
	Y_1_	0.11	0.10	0.13	0.03	0.94	0.09
	Y_2_	0.38	0.32	0.53	0.21	0.89	0.01
NPGA	X_1_	0.68	0.63	0.77	0.14	0.91	0.02
	X_2_	1.51	0.85	2.36	1.51	0.93	0.05
	X_3_	75.00	74.00	75.00	1.00	0.73	<0.001
	Y_1_	0.14	0.12	0.15	0.03	0.94	0.09
	Y_2_	0.73	0.67	0.80	0.13	0.95	0.16
NSGA	X_1_	0.72	0.67	0.76	0.09	0.96	0.27
	X_2_	2.14	0.97	3.15	2.18	0.92	0.03
	X_3_	65.00	65.00	66.00	1.00	0.60	<0.001
	Y_1_	0.11	0.09	0.29	0.05	0.96	0.23
	Y_2_	0.34	0.29	0.40	0.11	0.96	0.27
NSGA-II	X_1_	0.68	0.63	0.75	0.12	0.93	0.06
	X_2_	0.88	0.42	1.41	0.99	0.79	<0.001
	X_3_	75.00	74.00	75.00	1.00	0.66	<0.001
	Y_1_	0.15	0.14	0.17	0.03	0.94	0.11
	Y_2_	0.73	0.67	0.77	0.10	0.91	0.01

### 3.5 Experimental validation results

The Pareto non-inferior solution Scheme 2: X_1_ = 0.75, X_2_ = 0.35, X_3_ = 75% and Scheme 19: X_1_ = 0.68, X_2_ = 1.42, X_3_ = 75% obtained from NSGA-II search were selected and validated in three trials, the median and interquartile range values are shown in Table 6. The median levels of both Y_1_ and Y_2_ obtained by experimental validation were not statistically different from the target values obtained by NSGA-II search (*p* > 0.05). These results confirmed that the modeling and multi-objective optimization of this study were satisfactory. Therefore, both of these schemes can be used for lidocaine microemulsion preparation, and the preparation scheme according to desired preparation effects.

**TABLE 6:** Experimental validation results.

Response	Scheme 2			Scheme 19		
	Observed	Predicted	*p*	Observed	Predicted	*p*
Y_1_	0.15 ± 0.08	0.17	0.62	0.18 ± 0.07	0.14	0.23
Y_2_	0.81 ± 0.08	0.74	0.09	0.80 ± 0.14	0.80	1.00

## 4 Discussion

In this study, five multi-objective genetic algorithms, VEGA, MOGA, NPGA, NSGA, and NSGA-II, were used for multi-objective optimization of steady-state penetration rate and skin retention in lidocaine microemulsion preparations. We compared the optimization results of the five multi-objective genetic algorithms in terms of the most desirable optimization scheme and the search performance of the algorithms. Then, we experimentally validated that the modeling and optimization schemes proposed in this study work matched performance. A formulator focused on better transdermal properties may choose preparation conditions with a mass ratio of S/(O + S) of 0.75, a weight ratio of OL/(ALA + LA) of 0.35, and a water content of 75%. The resulting microemulsion shows a steady-state permeation rate of 0.17μg/(cm^2^·s) and skin retention of 0.74 mg/cm^2^. The corresponding optimal microemulsion formulation determined by this scheme was 1.11% OL, 0.64% ALA, 2.54% LA, 0.71% VES, 15.00% surfactant, 5.00% lidocaine, and 75.00% water. A formulator focused on preparing microemulsions with durations of action could choose a mass ratio of S/(O + S) of 0.68, a weight ratio of OL/(ALA + LA) of 1.42, and a water content of 75%. This results in a microemulsion with a steady-state permeation rate of 0.14μg/(cm^2^·s) and a skin retention of 0.80 mg/cm^2^. The corresponding optimal microemulsion formulation determined by this scheme was 3.23% OL, 0.45% ALA, 1.81% LA, 0.91% VES, 13.60% surfactant, 5.00% lidocaine, and 75.00% water.

Many studies have aimed to optimize formulations for microemulsions in the pharmaceutical and food industries. However, the transdermal permeation rates reported in the *in vitro* permeability experiments of these microemulsion formulations are not satisfactory compared to the permeation rates of Scheme 2 provided in this study. For example, Patel et al. conducted a study on the effect of different mixing ratios of excipients on the *in vitro* permeation of ketoconazole in a microemulsion, and found that the highest permeation rate of the optimal formula was 54.65 ± 1.72 μg/cm^2^/h (Patel et al., 2011). In an *in vitro* permeation study of a topical dosage form of hesperidin, Tsai et al. determined that the optimal microemulsion formulation had a permeation rate of 46.56 μg/cm^2^/h (Tsai et al., 2010). Wang et al. (2019) evaluated use of microemulsions for transdermal administration of high doses of lidocaine and prepared microemulsions for experimental evaluation. The permeability flux of the microemulsion formulation was determined 500.40 ± 23.34  μg/cm^2^/h. In contrast, our formulations showed a higher steady-state penetration rate of lidocaine microemulsion (0.17 μg/(cm^2^·s)), which was significantly higher than the penetration effect of the microemulsion formulations studied above. In our preparation targeted for longer duration of action, the penetration rate of lidocaine microemulsion reached 0.14 μg/(cm^2^·s)

Similarly, a few studies evaluating microemulsion preparation have explored the effect of microemulsion on skin retention. Maulvi et al. prepared a lidocaine tripotassium phosphate complex microemulsion. Skin retention studies showed that the microemulsion reached a maximum retention of 350 μg/g at 2 h (Maulvi et al., 2020). Boonme et al. evaluated the properties, stability, and skin permeability and retention of microemulsions containing nicotinamide. The 24 h nicotinamide infiltration into receptor fluids was 777.46 ± 60.11 μg/cm^2^ (Boonme et al., 2016). Niu et al. conducted a study on microemulsion-based keratin-chitosan gel to improve skin penetration/retention and activity of curcumin. The maximum skin drug retention was 3.75 ± 0.24 μg/cm^2^ for this microemulsion formulation (Niu et al., 2023). Scheme 19 in our study resulted in a longer duration of action of 0.80 mg/cm^2^. Which was higher than the skin retention in the above study. The skin retention in Scheme 2 reached 0.74 mg/cm^2^. Therefore, the regimens for preparation of lidocaine microemulsions proposed in this study performed well in terms of both penetration rate and skin retention.

Most microemulsion formulations contain more than 20% of surfactant. For example, Zhao, Jiang et al. studied a microemulsion preparation of Antarctic krill oil with 24% surfactant and 8% cosurfactant (Zhao et al., 2020). Wang et al. (2019) studied the feasibility of using microemulsions containing of 28% surfactants and cosurfactants for transdermal delivery of high-dose lidocaine. Ngawhirunpat et al. (2013) studied the preparation of ketoprofen microemulsions for transdermal delivery. Among the formulations that achieved the highest skin permeation flux, the dosage of surfactant (Cremophor RH40) was 22.5%, and the dosage of cosurfactant (PEG 400) was 22.5%. Xu et al. (2016) prepared microalgae oil microemulsions without co-surfactants, but but with more than 20% surfactant. These studies show that many microemulsion formulations rely on higher amounts of surfactant and cosurfactant. In our study, Scheme 2 and Scheme 19 had surfactant content below 15% and as low as 13.6%. This result demonstrates our progress in reducing the amount of surfactant and cosurfactant in microemulsion formulations, which should reduce toxicity and skin irritation.

In conclusion, the preparation schemes proposed in this study were practical and improved upon previously developed formulations using an optimization algorithm. In addition, NSGA-II greatly reduced the number of pre-experiments necessary to optimize preparation conditions, resulted in reduced use of materials and significant cost savings. Furthermore, our study demonstrated the value of using a multi-objective optimization strategy in the pharmaceutical field.

## 5 Conclusion

This study proposed two sets of lidocaine microemulsion preparation. The first included a mass ratio of surfactant/(oil phase + surfactant) (X_1_) = 0.75, a mass ratio of olive oil/(α-linolenic acid + linoleic acid) (X_2_) = 0.35, and water content W% (X_3_) = 75%, which resulted in better transdermal performance. When X_1_ = 0.68, X_2_ = 1.42, and X_3_ = 75%, the microemulsion resulted in longer anesthesia duration.

## Data Availability

The original contributions presented in the study are included in the article/Supplementary Material, further inquiries can be directed to the corresponding author.

## Abbreviations

ALA, α-linolenic acid; IQR, Interquartile-quartile range; LA, Linoleic acid; MOGA, Multiple Objective Genetic Algorithm; NPGA, Niched Pareto Genetic Algorithm; NSGA, Non-dominated Sorting Genetic Algorithm; NSGA-Ⅱ, Non-dominated Sorting Genetic Algorithm-II; OL, Olive oil; VES, Vitamin E succinate; VEGA, Vector Evaluated Genetic Algorithm.
