# Supplementary material for: A study on the preparation conditions of lidocaine microemulsion based on multi-objective genetic algorithm
Source: Front Pharmacol. 2023 Sep 29;14:1272454. doi: 10.3389/fphar.2023.1272454 (PMC10576434; doi:10.3389/fphar.2023.1272454)
Supplement: Supplementary file 1 [file DataSheet1.docx]

The algorithm flowcharts of the five multi-objective genetic algorithms are shown in Figures S1-S5, respectively.


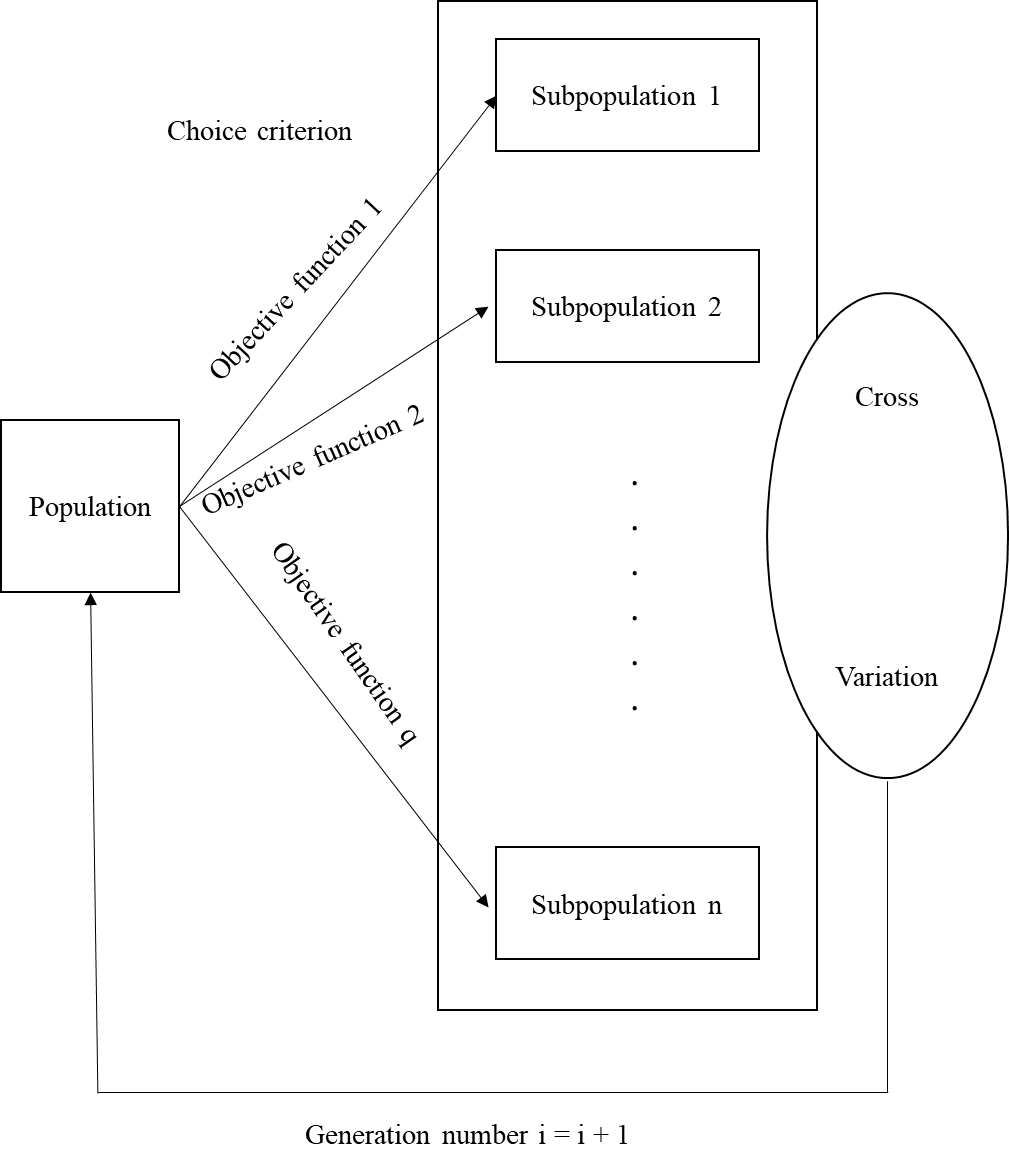


**Figure S1** VEGA algorithm flow chart


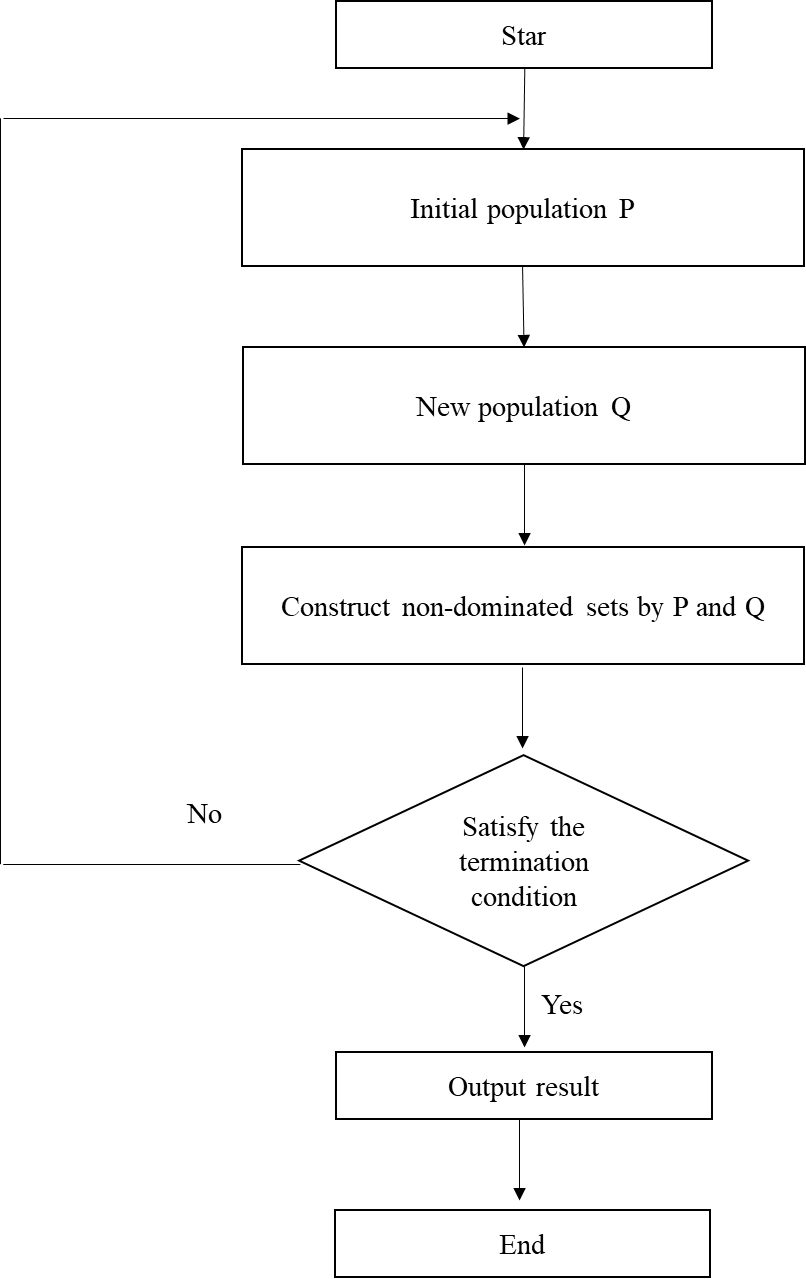


**Figure S2** MOGA algorithm flow chart


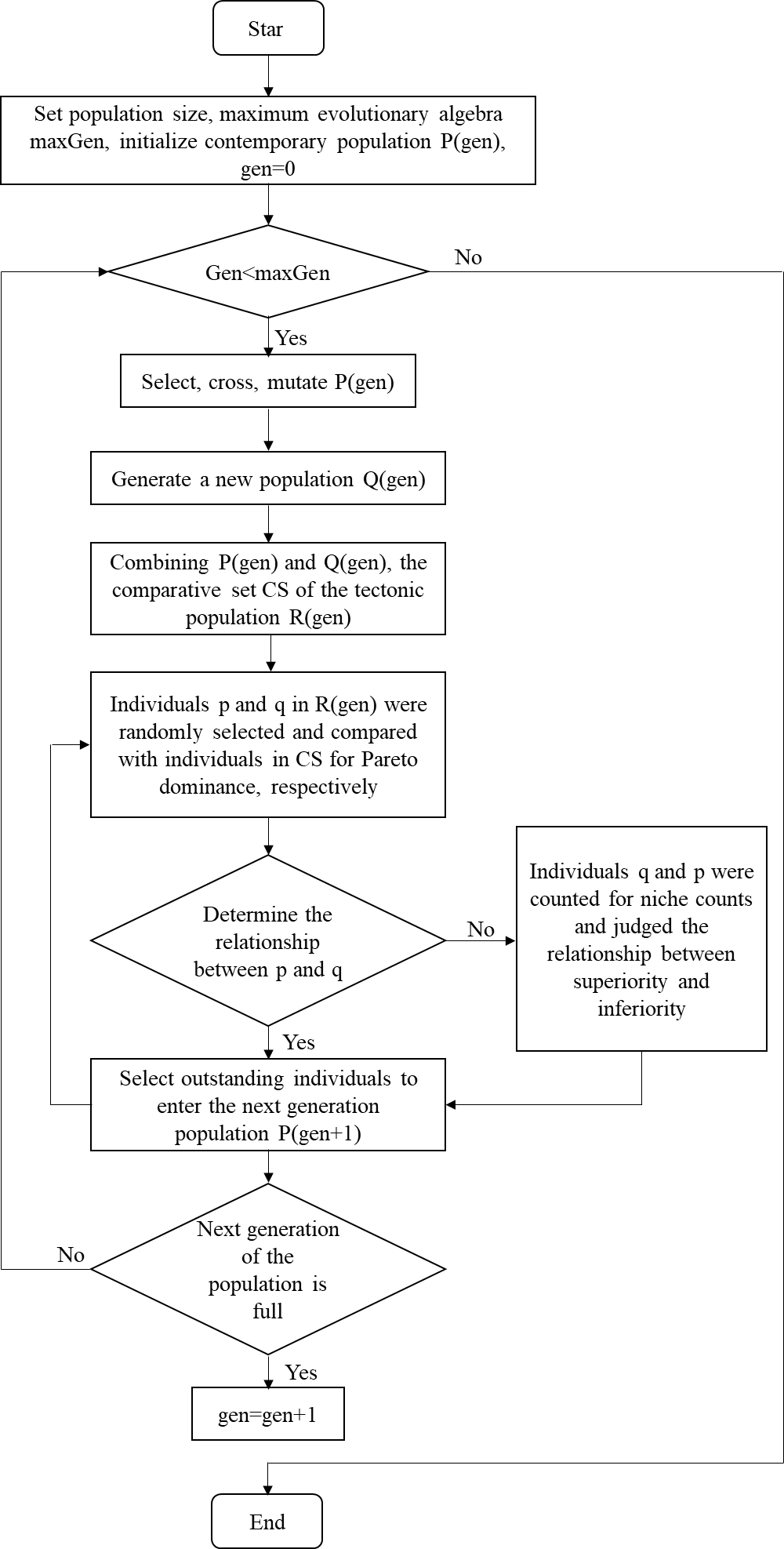


**Figure S3** NPGA algorithm flow chart


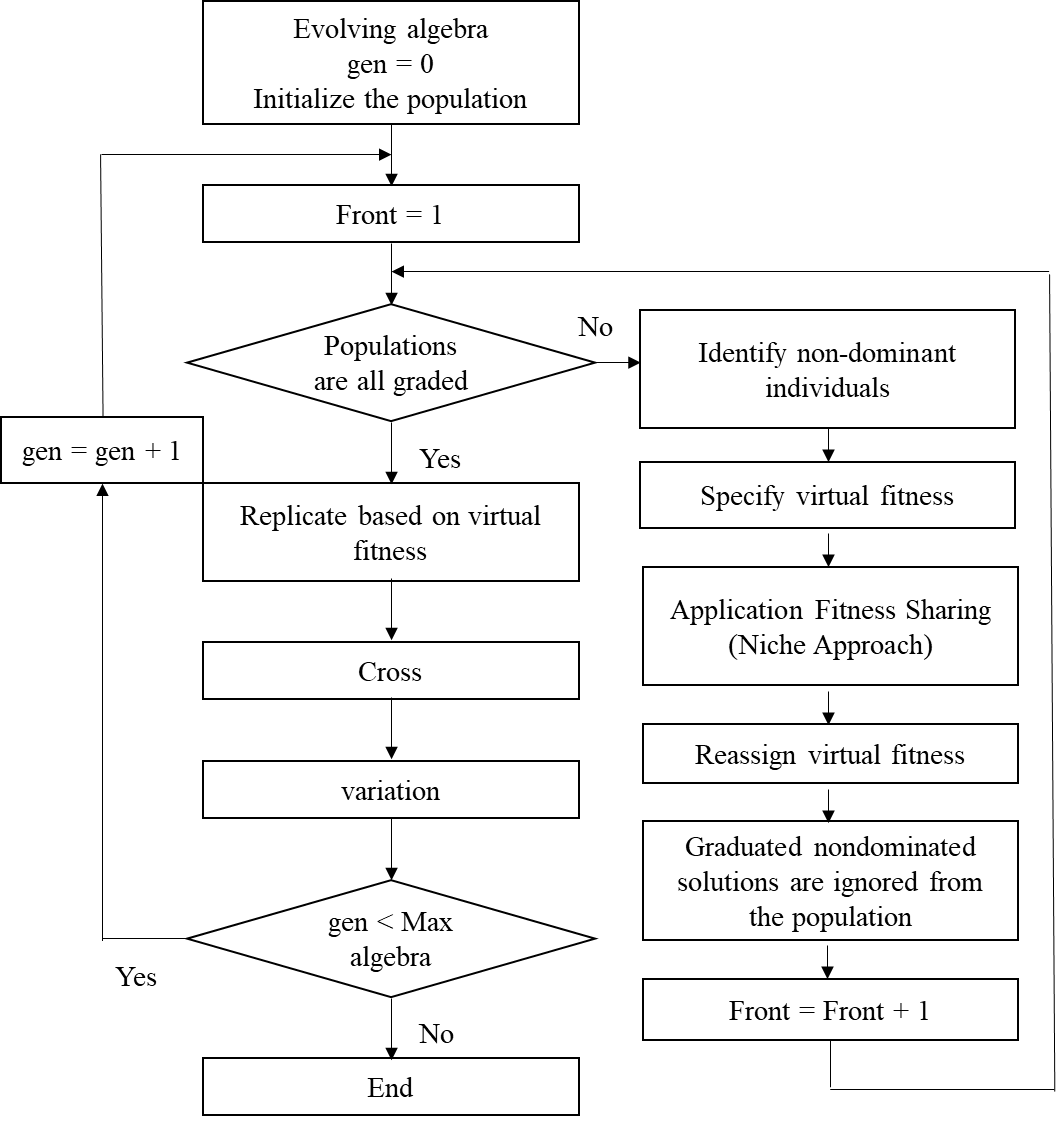


**Figure S4** NSGA algorithm flow chart


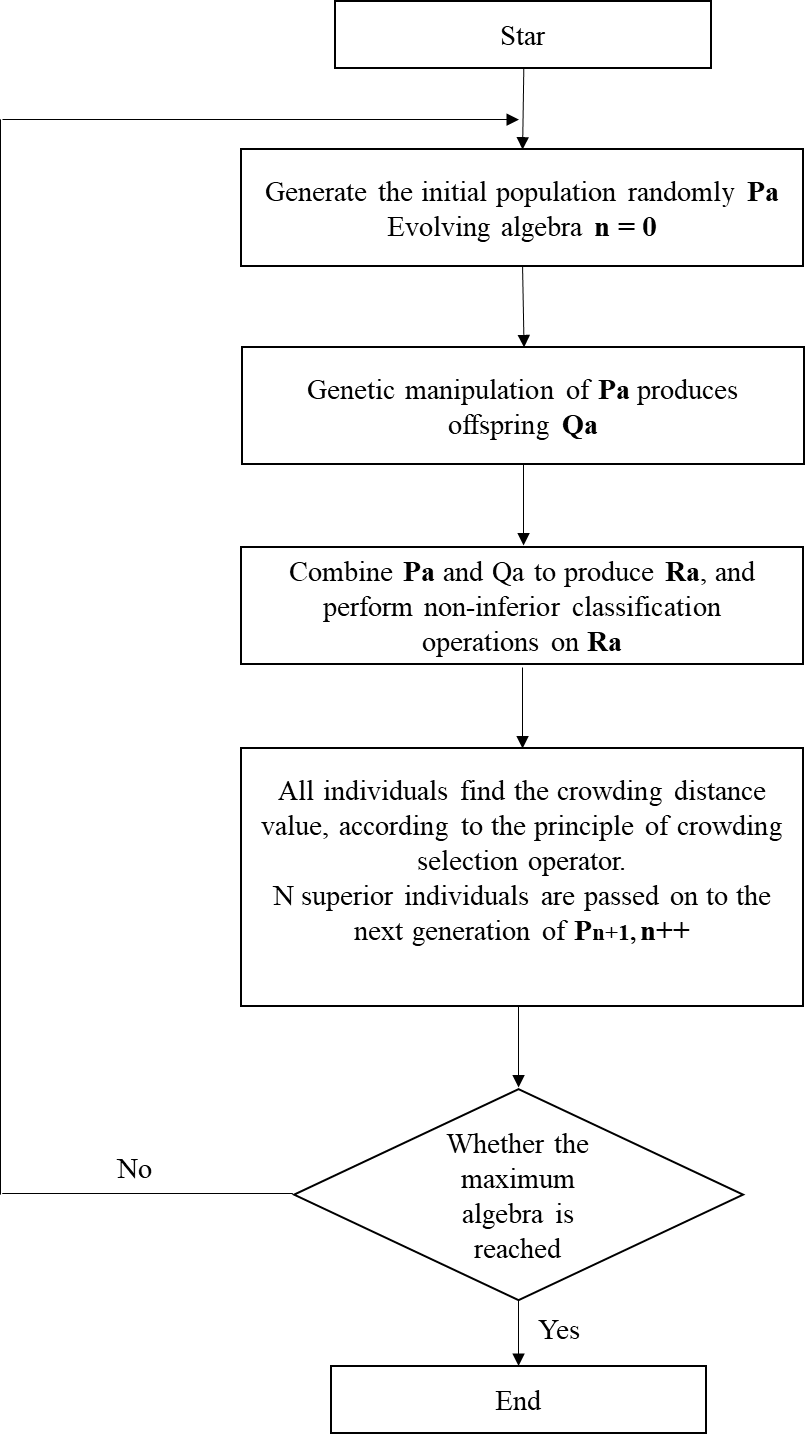


**Figure S5** NSGA-Ⅱ algorithm flow chart

The non-inferior solution schemes obtained from the VEGA random search 30 times are shown in Table S1. According to the requirements for the objective function, the optimum process conditions were selected in the Pareto solution set with X_1_ = 0.65, X_2_ = 0.83, and X_3_ = 75% for the scheme 29, and the steady-state permeation rate and skin retention of lidocaine microemulsion were obtained as Y_1_ = 0.15g/(cm^2^·s) and Y_2_ = 0.75 mg/cm^2^. The maximum fitness and average fitness evolution curves of the two sub-objective functions obtained by VEGA optimization are shown in Fig. S6 and Fig. S7. As seen from the figure, the maximum fitness and average fitness of VEGA reach stability and good evolutionary performance after 9 generations of evolution.

**TableS1** Random search results of VEGA

| Solutions | Pareto optimal solution set | | | Response | | Surfactant (%) |
| --- | --- | --- | --- | --- | --- | --- |
|  | X_1_ | X_2_ | X_3_(%) | Y_1_(g/(cm^2^·s)) | Y_2_(mg/cm^2^) |  |
| 1 | 0.78 | 0.77 | 75 | 0.16 | 0.73 | 15.6 |
| 2 | 0.67 | 0.51 | 75 | 0.16 | 0.73 | 13.4 |
| 3 | 0.67 | 1.27 | 75 | 0.14 | 0.78 | 13.4 |
| 4 | 0.70 | 0.37 | 75 | 0.17 | 0.69 | 14.0 |
| 5 | 0.72 | 1.17 | 75 | 0.14 | 0.74 | 14.4 |
| 6 | 0.66 | 1.84 | 74 | 0.13 | 0.73 | 13.9 |
| 7 | 0.69 | 0.40 | 75 | 0.17 | 0.72 | 13.8 |
| 8 | 0.71 | 1.12 | 75 | 0.14 | 0.73 | 14.2 |
| 9 | 0.66 | 0.63 | 72 | 0.13 | 0.44 | 15.2 |
| 10 | 0.67 | 2.27 | 74 | 0.12 | 0.77 | 14.1 |
| 11 | 0.70 | 0.63 | 75 | 0.16 | 0.70 | 14.0 |
| 12 | 0.74 | 0.52 | 74 | 0.16 | 0.65 | 15.5 |
| 13 | 0.60 | 2.00 | 75 | 0.13 | 0.80 | 12.0 |
| 14 | 0.79 | 1.38 | 75 | 0.14 | 0.75 | 15.8 |
| 15 | 0.72 | 1.60 | 74 | 0.13 | 0.72 | 15.1 |
| 16 | 0.75 | 1.18 | 74 | 0.14 | 0.67 | 15.8 |
| 17 | 0.62 | 1.05 | 75 | 0.15 | 0.74 | 12.4 |
| 18 | 0.73 | 0.63 | 74 | 0.16 | 0.67 | 15.3 |
| 19 | 0.62 | 1.58 | 74 | 0.13 | 0.66 | 13.0 |
| 20 | 0.74 | 0.62 | 75 | 0.16 | 0.73 | 14.8 |
| 21 | 0.67 | 1.87 | 74 | 0.12 | 0.64 | 14.1 |
| 22 | 0.60 | 0.39 | 74 | 0.16 | 0.62 | 12.6 |
| 23 | 0.70 | 1.42 | 74 | 0.13 | 0.67 | 14.7 |
| 24 | 0.75 | 1.09 | 75 | 0.15 | 0.74 | 15.0 |
| 25 | 0.71 | 1.11 | 75 | 0.14 | 0.73 | 14.2 |
| 26 | 0.71 | 3.81 | 74 | 0.11 | 0.75 | 14.9 |
| 27 | 0.79 | 3.27 | 74 | 0.11 | 0.77 | 16.6 |
| 28 | 0.75 | 1.13 | 74 | 0.14 | 0.64 | 15.8 |
| **29** | **0.65** | **0.83** | **75** | **0.15** | **0.75** | **13.0** |
| 30 | 0.64 | 1.72 | 75 | 0.13 | 0.80 | 12.8 |

**Note**: Bold indicates the most ideal solution for this method


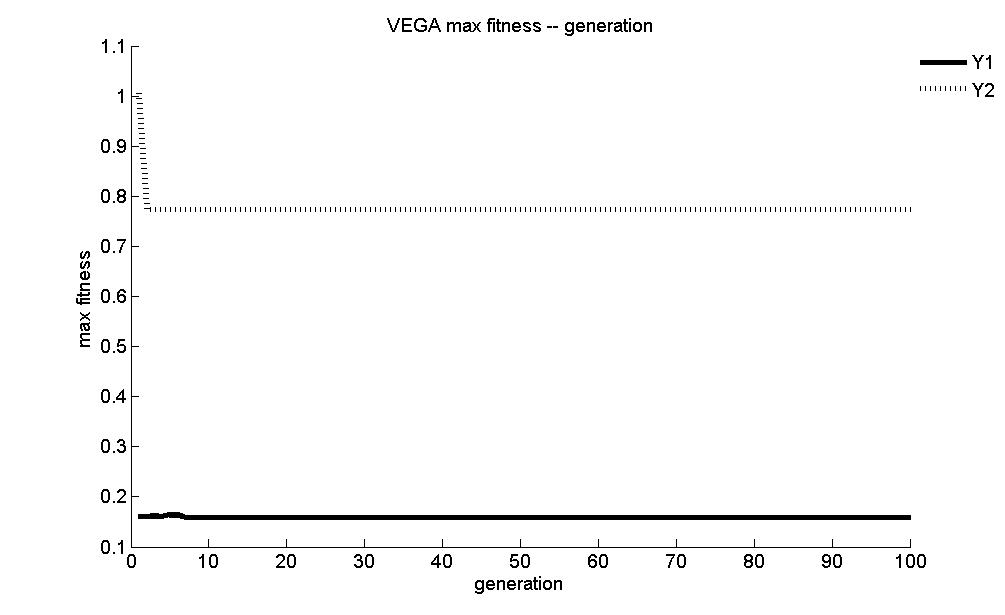


**Figure S6** VEGA maximum adaptation evolutionary curve


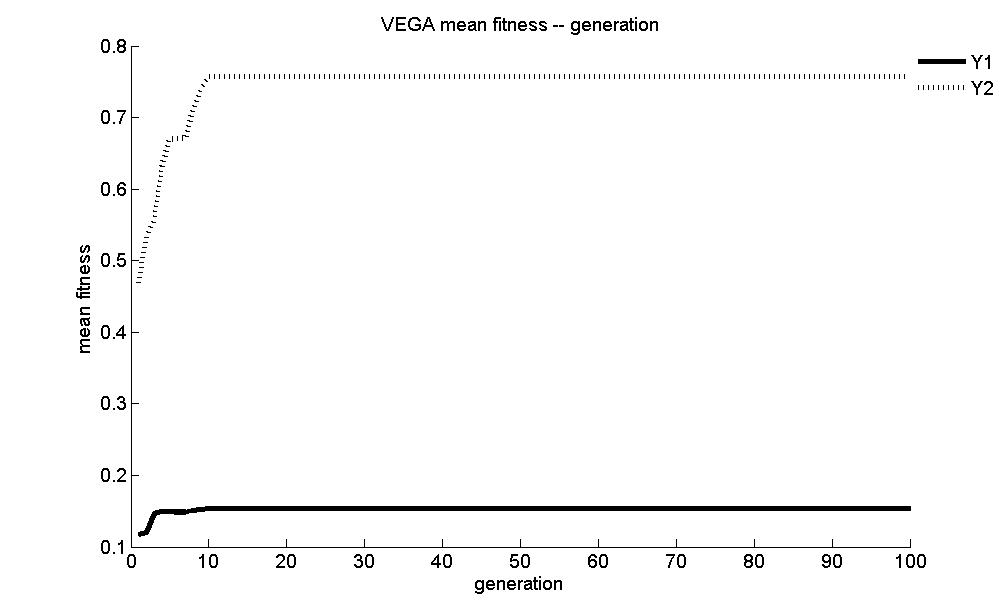


**Figure S7** VEGA average adaptation evolutionary curve

The non-inferior solution schemes obtained from the MOGA random search 30 times are shown in Table S2. According to the requirements for the objective function, the optimum process conditions were selected in the Pareto solution set with X_1_ = 0.75, X_2_ = 0.99, and X_3_ = 75% for the scheme 6, and the steady-state permeation rate and skin retention of lidocaine microemulsion were obtained as Y_1_ = 0.15g/(cm^2^·s) and Y_2_ = 0.70 mg/cm^2^. The maximum fitness and average fitness evolution curves of the two sub-objective functions obtained by MOGA optimization are shown in Fig. S8 and Fig. S9. As seen from the figure, the maximum fitness and average fitness of MOGA reach stability and good evolutionary performance after 11 generations of evolution.

**TableS2** Random search results of MOGA

| Solutions | Pareto optimal solution set | | | Response | | Surfactant (%) |
| --- | --- | --- | --- | --- | --- | --- |
|  | X_1_ | X_2_ | X_3_(%) | Y_1_(g/(cm^2^·s)) | Y_2_(mg/cm^2^) |  |
| 1 | 0.80 | 3.89 | 71 | 0.11 | 0.30 | 19.2 |
| 2 | 0.71 | 0.73 | 67 | 0.13 | 0.32 | 19.9 |
| 3 | 0.64 | 1.92 | 75 | 0.13 | 0.77 | 12.8 |
| 4 | 0.78 | 0.90 | 71 | 0.14 | 0.36 | 18.7 |
| 5 | 0.66 | 2.36 | 72 | 0.10 | 0.52 | 15.2 |
| **6** | **0.75** | **0.99** | **75** | **0.15** | **0.70** | **15.0** |
| 7 | 0.61 | 3.91 | 70 | 0.09 | 0.41 | 15.3 |
| 8 | 0.65 | 1.00 | 68 | 0.10 | 0.31 | 17.6 |
| 9 | 0.71 | 2.84 | 73 | 0.11 | 0.61 | 15.6 |
| 10 | 0.60 | 2.30 | 75 | 0.13 | 0.81 | 12.0 |
| 11 | 0.63 | 0.99 | 72 | 0.12 | 0.44 | 14.5 |
| 12 | 0.67 | 0.54 | 71 | 0.13 | 0.38 | 16.1 |
| 13 | 0.80 | 4.00 | 72 | 0.10 | 0.49 | 18.4 |
| 14 | 0.74 | 0.59 | 66 | 0.14 | 0.34 | 21.5 |
| 15 | 0.69 | 0.26 | 73 | 0.15 | 0.47 | 15.2 |
| 16 | 0.68 | 3.61 | 71 | 0.09 | 0.42 | 16.3 |
| 17 | 0.65 | 3.05 | 69 | 0.09 | 0.33 | 16.9 |
| 18 | 0.75 | 0.40 | 68 | 0.15 | 0.28 | 20.3 |
| 19 | 0.72 | 2.44 | 73 | 0.11 | 0.57 | 15.8 |
| 20 | 0.72 | 2.03 | 66 | 0.11 | 0.30 | 20.9 |
| 21 | 0.73 | 2.77 | 70 | 0.10 | 0.31 | 18.3 |
| 22 | 0.77 | 3.30 | 71 | 0.10 | 0.34 | 18.5 |
| 23 | 0.76 | 2.00 | 74 | 0.12 | 0.66 | 16.0 |
| 24 | 0.75 | 1.31 | 72 | 0.13 | 0.42 | 17.3 |
| 25 | 0.63 | 2.83 | 66 | 0.08 | 0.35 | 18.3 |
| 26 | 0.62 | 2.29 | 67 | 0.08 | 0.33 | 17.4 |
| 27 | 0.79 | 2.72 | 68 | 0.11 | 0.20 | 21.3 |
| 28 | 0.71 | 2.41 | 74 | 0.11 | 0.65 | 14.9 |
| 29 | 0.65 | 3.72 | 69 | 0.09 | 0.37 | 16.9 |
| 30 | 0.69 | 3.43 | 66 | 0.10 | 0.32 | 20.0 |

**Note**: Bold indicates the most ideal solution for this method


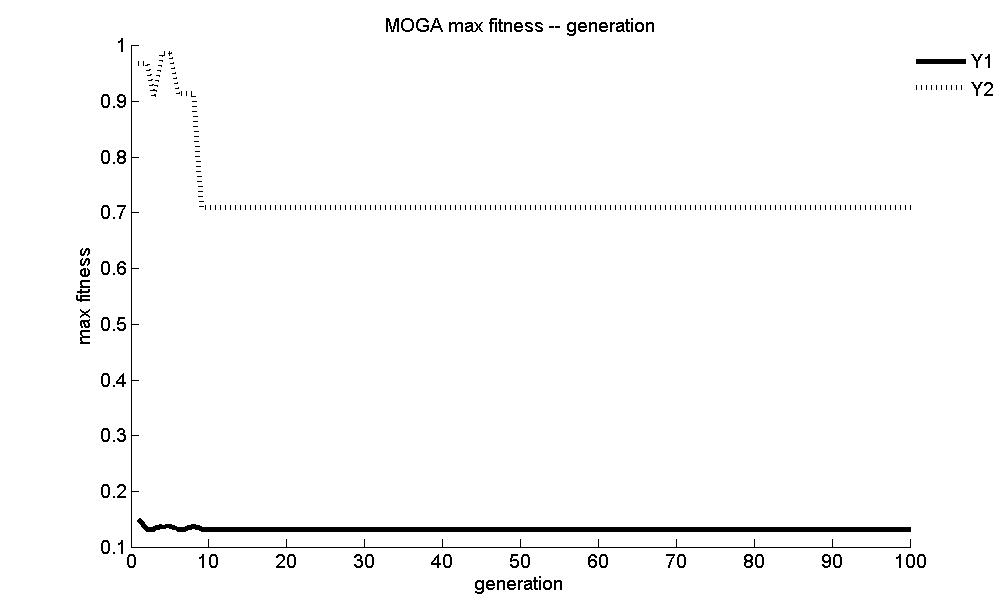


**Figure S8** MOGA maximum adaptation evolutionary curve


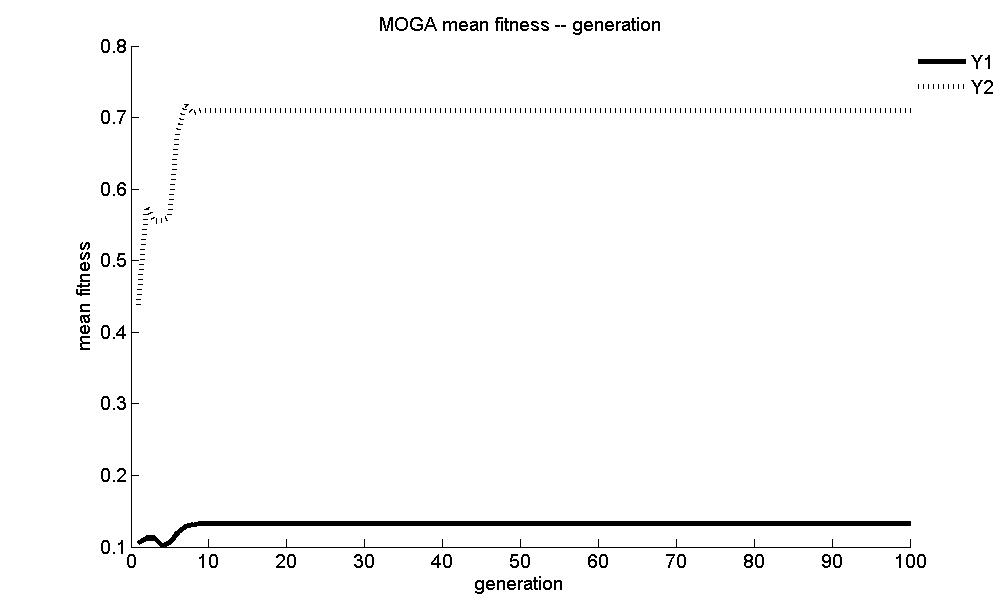


**Figure S9** MOGA average adaptation evolutionary curve

The non-inferior solution schemes obtained from the NSGA random search 30 times are shown in Table S3. According to the requirements for the objective function, the optimum process conditions were selected in the Pareto solution set with X_1_ = 0.71, X_2_ = 0.29, and X_3_ = 65% for the scheme 1, and the steady-state permeation rate and skin retention of lidocaine microemulsion were obtained as Y_1_ = 0.14g/(cm^2^·s) and Y_2_ = 0.42 mg/cm^2^. The maximum fitness and average fitness evolution curves of the two sub-objective functions obtained by NSGA optimization are shown in Fig. S10 and Fig. S11. As seen from the figure, the maximum fitness and average fitness of NSGA reach stability and good evolutionary performance after 7 generations of evolution.

**TableS3** Random search results of NSGA

| Solutions | Pareto optimal solution set | | | Response | | Surfactant (%) |
| --- | --- | --- | --- | --- | --- | --- |
|  | X_1_ | X_2_ | X_3_ | Y_1_(g/(cm^2^·s)) | Y_2_(mg/cm^2^) |  |
| **1** | **0.71** | **0.29** | **65** | **0.14** | **0.42** | **21.3** |
| 2 | 0.72 | 0.56 | 65 | 0.13 | 0.40 | 21.6 |
| 3 | 0.80 | 3.77 | 66 | 0.13 | 0.16 | 23.2 |
| 4 | 0.70 | 1.26 | 65 | 0.12 | 0.40 | 21.0 |
| 5 | 0.67 | 1.54 | 66 | 0.10 | 0.34 | 19.4 |
| 6 | 0.73 | 3.46 | 65 | 0.11 | 0.29 | 21.9 |
| 7 | 0.60 | 2.72 | 65 | 0.07 | 0.40 | 18.0 |
| 8 | 0.69 | 2.39 | 66 | 0.10 | 0.33 | 20.0 |
| 9 | 0.74 | 2.28 | 65 | 0.11 | 0.32 | 22.2 |
| 10 | 0.79 | 0.31 | 66 | 0.17 | 0.29 | 22.9 |
| 11 | 0.66 | 2.00 | 65 | 0.09 | 0.39 | 19.8 |
| 12 | 0.68 | 2.60 | 66 | 0.09 | 0.33 | 19.7 |
| 13 | 0.64 | 3.41 | 66 | 0.08 | 0.36 | 18.6 |
| 14 | 0.60 | 3.88 | 65 | 0.08 | 0.41 | 18.0 |
| 15 | 0.74 | 2.92 | 66 | 0.11 | 0.25 | 21.5 |
| 16 | 0.77 | 0.53 | 65 | 0.16 | 0.37 | 23.1 |
| 17 | 0.69 | 3.52 | 66 | 0.09 | 0.30 | 20.0 |
| 18 | 0.69 | 1.23 | 65 | 0.11 | 0.38 | 20.7 |
| 19 | 0.80 | 1.11 | 65 | 0.16 | 0.31 | 24.0 |
| 20 | 0.75 | 3.11 | 65 | 0.11 | 0.27 | 22.5 |
| 21 | 0.65 | 1.75 | 65 | 0.09 | 0.41 | 19.5 |
| 22 | 0.79 | 0.29 | 65 | 0.18 | 0.36 | 23.7 |
| 23 | 0.67 | 3.07 | 66 | 0.09 | 0.33 | 19.4 |
| 24 | 0.71 | 3.62 | 66 | 0.10 | 0.29 | 20.6 |
| 25 | 0.72 | 0.47 | 65 | 0.14 | 0.41 | 21.6 |
| 26 | 0.74 | 1.40 | 65 | 0.13 | 0.37 | 22.2 |
| 27 | 0.65 | 0.56 | 65 | 0.11 | 0.46 | 19.5 |
| 28 | 0.76 | 1.45 | 65 | 0.14 | 0.34 | 22.8 |
| 29 | 0.80 | 3.25 | 65 | 0.13 | 0.19 | 24.0 |
| 30 | 0.79 | 2.31 | 65 | 0.14 | 0.25 | 23.7 |

**Note**: Bold indicates the most ideal solution for this method


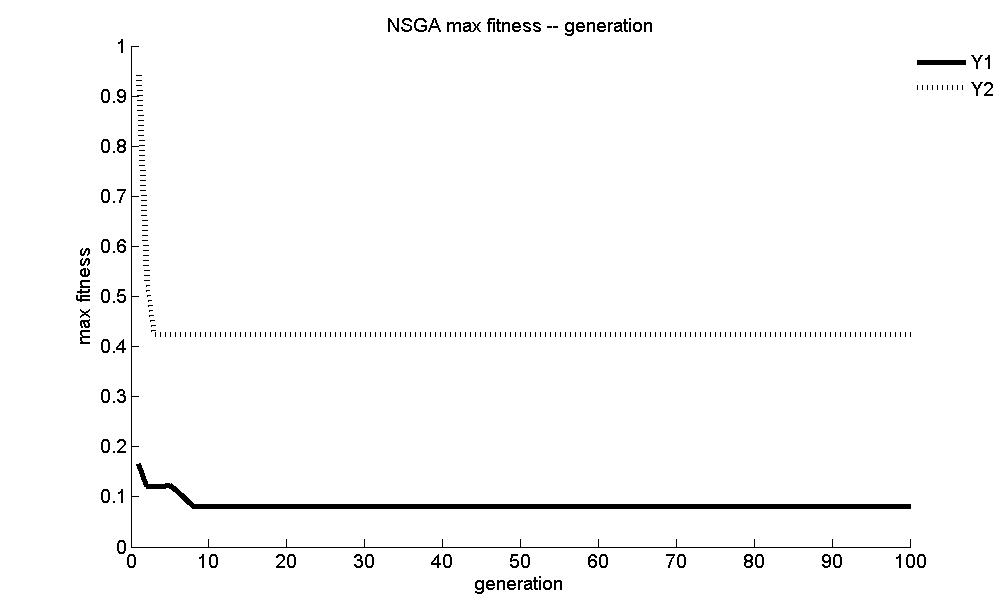


**Figure S10** NSGA maximum adaptation evolutionary curve


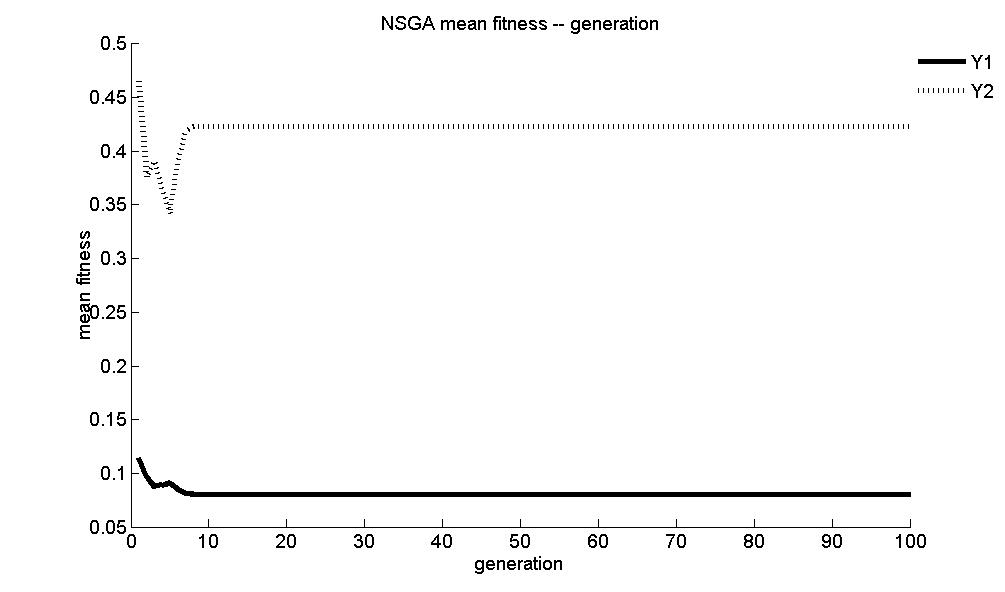


**Figure S11** NSGA average adaptation evolutionary curve
